# Corticosteroid Injections and Risk of Fracture

**DOI:** 10.1001/jamanetworkopen.2024.14316

**Published:** 2024-05-31

**Authors:** Terin T. Sytsma, Shannon Thomas, Karen M. Fischer, Laura S. Greenlund

**Affiliations:** 1 Division of Community Internal Medicine, Geriatrics, and Palliative Care, Mayo Clinic, Rochester, Minnesota; 2 University of Minnesota, Minneapolis; 3 Division of Clinical Statistics, Mayo Clinic, Rochester, Minnesota

## Abstract

**Importance:**

Corticosteroid injections (CSIs) are an important tool for pain relief in many musculoskeletal conditions, but the longitudinal effects of these treatments on bone health and fracture risk are unknown.

**Objective:**

To determine whether cumulative doses of corticosteroid injections are associated with higher risk of subsequent osteoporotic and nonosteoporotic fractures.

**Design, Setting, and Participants:**

This cohort study included adult patients receiving any CSI from May 1, 2018, through July 1, 2022. Eligible patients resided in Olmsted County, Minnesota, and were empaneled to receive primary care within the Mayo Clinic. Cox proportional hazards regression models were used to evaluate risk of fracture based on cumulative injected corticosteroid dose.

**Exposure:**

Receipt of any CSI during the study period.

**Main Outcomes and Measures:**

The primary outcome was risk of fracture by total triamcinolone equivalents received. Secondary outcomes consisted of risks of fracture based on triamcinolone equivalents received in subgroups of patients not at high risk for fracture and patients with osteoporosis.

**Results:**

A total of 7197 patients were included in the study (mean [SD] age, 64.4 [14.6] years; 4435 [61.6%] women; 183 [2.5%] Black and 6667 [92.6%] White), and 346 (4.8%) had a new fracture during the study period. Of these fractures, 149 (43.1%) were considered osteoporotic. In the adjusted Cox proportional hazards regression model, there was no association of higher fracture risk based on cumulative CSI dose (adjusted hazard ratio [HR], 1.04 [95% CI, 0.96-1.11]). There was also no associated higher risk of fracture in the non–high-risk (adjusted HR, 1.11 [95% CI, 0.98-1.26]) or osteoporosis (adjusted HR, 1.01 [95% CI, 0.90-1.11]) subgroups. Age, Charleson Comorbidity Index, and previous fracture were the only factors that were associated with higher fracture risk.

**Conclusions and Relevance:**

In this cohort study of cumulative injected corticosteroid dose and risk of subsequent fracture, no association was observed, including in patients with a preexisting diagnosis of osteoporosis. Treatment of painful conditions with CSI should not be withheld or delayed owing to concern about fracture risk.

## Key Points

**Question:**

Are high numbers of corticosteroid injections (CSIs) associated with subsequent fractures?

**Findings:**

In this cohort study of 7197 patients over a 4-year period, higher fracture risk was not associated with the cumulative dose of injected corticosteroid, regardless of osteoporosis status.

**Meaning:**

Corticosteroid injections are an important option for pain treatment, and clinicians should not withhold CSI owing to fear of fracture risk.

## Teaser

This cohort study assesses whether cumulative doses of corticosteroid injections are associated with risk of fracture in adults.

## Introduction

A key strategy in management of musculoskeletal pain, especially in elderly patients, involves joint and bursae corticosteroid injections (CSIs). Typically, a water-insoluble crystalline corticosteroid is used to prolong presence within the injected site and to limit absorption. However, some systemic absorption of injected corticosteroids occurs, potentially placing patients at risk for adverse effects of corticosteroids.^1,2,3^ Unlike oral corticosteroids, the extent and clinical significance of the systemic effects of CSIs are largely unknown, and there is no established dose or frequency threshold for safety of CSIs. Specific dose information has been difficult to determine because a patient may receive CSIs in multiple clinical settings (eg, primary care, orthopedics, rheumatology, radiology, sports medicine), and there is no prescription associated with the medication.

Osteoporosis affects approximately 10 million Americans, and 1 in 2 women and 1 in 5 men older than 50 years will have an osteoporotic fracture during their lifetime.^4^ In the case of oral corticosteroids, large retrospective cohort studies and meta-analyses have established that dosages greater than 7.5 mg/d may be associated with increased fracture risk,^5,6,7^ and there is conflicting evidence as to whether bone density in inflammatory rheumatic diseases is affected at even lower oral doses.^8,9^ Van Staa et al^6^ showed that increased fracture risk began within 3 months of commencing oral corticosteroid therapy and was related to higher daily and cumulative dose.^7^ Patients taking a long-term dosage of prednisolone of at least 7.5 mg/d were significantly more likely to sustain a fracture compared with those receiving a low daily dosage (<2.5 mg/d).^7^ In a meta-analysis of patients with rheumatoid arthritis, lower dosage use (≤7.5 mg/d) was associated with few adverse events.^10^ Studies assessing fracture risk associated with epidural CSIs to treat low back pain have been conflicting, with 1 large retrospective study showing an association^11^ and other smaller studies showing none.^12,13,14^

To better understand the fracture risk associated with CSIs, we conducted a cohort study of patients receiving any CSI in all possible clinical settings over a 4-year span beginning in 2018. Our primary outcome was risk of fracture by total number of injected triamcinolone equivalents received. Secondary outcomes were risks of fracture by total triamcinolone equivalents in subgroups of patients not at high risk for fracture and in patients with osteoporosis. We hypothesized that higher fracture risk would be associated with increasing cumulative injected triamcinolone equivalent doses.

## Methods

In this cohort study of primary care patients, we used an institutional electronic health record database to identify adults (aged ≥18 years) residing in Olmsted County, Minnesota, and empaneled to receive primary care within the Mayo Clinic who received any CSI beginning May 1, 2018, and ending July 1, 2022. The Mayo Clinic Institutional Review Board deemed this study exempt from review and the need for informed consent because it was not considered human participant research. We followed the Strengthening the Reporting of Observational Studies in Epidemiology (STROBE) reporting guideline.

### Study Participants 

*Current Procedural Terminology* codes (eTable 1 in Supplement 1) and medications injected (methylprednisolone, triamcinolone, betamethasone, or dexamethasone) were used to identify patients receiving CSIs. Calculation of CSI corticosteroid equivalents used the following : 1 mg of methylprednisolone = 1 mg of triamcinolone = 0.2 mg of betamethasone = 0.2 mg of dexamethasone.^15^ Charlson Comorbidity Index was used to quantify comorbidities and collected using codes from the *International Classification of Diseases, Ninth Revision* (*ICD-9*) and *International Statistical Classification of Diseases, Tenth Revision* (*ICD-10*).^16^ Oral corticosteroid use was determined by prescriptions issued between May 1, 2017, and July 1, 2022, and total dose was calculated using the following conversion doses: 1 mg of prednisone = 1 mg of prednisolone = 0.15 mg of dexamethasone = 4.0 mg of hydrocortisone = 0.8 mg of methylprednisolone. Patients with prednisone use of no more than 2.5 mg/d for longer than 30 days and more than 2.5 mg/d for 30 or fewer days were included in the study. Patients were excluded if they had a prescription for oral prednisone equivalents greater than 2.5 mg/d for more than 30 days.

Initial encounter codes (*ICD-9* and *ICD-10*) for fractures of the limbs, spine, pelvis, and ribs and stress fractures were used to identify events, and a specific subset of these (vertebral, wrist, and hip) were classified as osteoporotic fractures (eTable 2 in Supplement 1). Fractures occurring between May 1, 2012, and April 30, 2018, were considered previous fractures, and those occurring on or after May 1, 2018, were included in the analysis for the study period. Fractures were only included in the analysis if they occurred after the first CSI administration.

For subgroup analysis, we identified 2 subgroups. The first subgroup included patients who were considered not at high risk for fractures, that is, having no preexisting diagnosis of osteoporosis or inflammatory arthritis such as rheumatoid arthritis. The second subgroup included patients with a diagnosis of osteoporosis prior to the start of the study period.

### Statistical Analysis

Analyses were performed for 3 different datasets. One dataset contained the whole group, and the additional datasets were the non–high-risk and the osteoporosis subgroups. Demographic characteristics for each group were reported using mean (SD or range) for continuous variables and frequencies for categorical variables.

Differences in injected corticosteroid level were analyzed via a Cox proportional hazards regression model. The assumption of proportional hazards was tested and confirmed by plotting Schoenfeld residuals. Patients received injections past their initial encounter, which was considered using a time-varying covariate for injected corticosteroid level. This variable was additive and did not reset over time. Other covariates that were adjusted for were demographic factors associated with fracture risk (age at initial injection, sex, race and ethnicity [Asian, Black, White, or other (including African, American Indian or Alaska Native, Chamorro, Guamanian, and Samoan)]); Charlson Comorbidity Index, which is a composite of a patient’s general health status taking into account multiple comorbidities^16^; number of CSIs prior to the study period; and previous fracture (2012-2018). Data on race and ethnicity were collected for this study because these factors have been shown to alter fracture risk in the US population.^17^ Self-reported lifestyle factors such as current smoking and alcohol use multiple times a week were evenly distributed between the cumulative dose quartiles, as was body mass index of less than 20 (calculated as weight in kilograms divided by height in meters squared) (eTable 3 in Supplement 1). Death was treated as a competing event using a cause-specific hazards model, which censored the patient on the date of death such that if a fracture came before death, that patient would be stopped at the time of the fracture.

Hazard ratios (HRs) and 95% CIs are reported. Two-sided *P* < .05 was considered significant. Statistical analysis was performed using SAS statistical software, version 9.4 (SAS Institute Inc).

## Results

A total of 7197 patients were included in the study (mean [SD] age, 64.4 [14.6] years; 4435 women [61.6%] and 2762 [38.4%] men). In terms of race and ethnicity, 174 patients (2.4%) were Asian, 183 (2.5%) were Black, 6667 (92.6%) were White, and 102 (1.4%) were of other race or ethnicity. The mean (SD) Charlson Comorbidity Index was 1.1 (1.9 [range, 0-19]). The mean (SD) total cumulative CSI dose during the study period was 141.8 (159.0 [range, 2.7-2140.3]) mg of triamcinolone equivalents (Table 1). Of the 33 684 total CSIs given, most patients received CSIs in large joints (n = 15 040), with the spine facet as the next most common CSI location (n = 6356) (Table 2).

**Table 1.** Demographic Characteristics of the Study Cohort

Characteristic	Patient group^a^		
	All (N = 7197)	Non–high-risk (n = 4741)	Osteoporosis (n = 1845)
Age, y			
Mean (SD)	64.4 (14.6)	61.0 (14.2)	73.8 (11.5)
Median (range)	65.0 (18-102)	61.0 (18-100)	75.0 (30-102)
Sex			
Women	4435 (61.6)	2552 (53.8)	1540 (83.5)
Men	2762 (38.4)	2189 (46.2)	305 (16.5)
Race and ethnicity			
Asian	174 (2.4)	117 (2.5)	37 (2.0)
Black	183 (2.5)	127 (2.7)	37 (2.0)
White	6667 (92.6)	4371 (92.2)	1742 (94.4)
Other^b^	102 (1.4)	75 (1.6)	15 (0.8)
Unknown	71 (1.0)	51 (1.1)	14 (0.8)
Charlson Comorbidity Index			
Mean (SD)	1.1 (1.9)	0.9 (1.7)	1.5 (2.10)
Median (range)	0 (0-19)	0 (0-15)	1.0 (0-17)
No. of past injections			
Mean (SD)	2.9 (3.6)	2.5 (3.1)	4.0 (4.5)
Median (range)	2.0 (0-40)	1.0 (0-32)	3.0 (0-40)
Total CSI dose, mg^c^			
Mean (SD)	141.8 (159.0)	130.9 (143.9)	164.0 (182.5)
Median (range)	80.0 (2.7-2140.3)	80.0 (4.0-2000.0)	90.0 (2.7-2140.3)
Fracture	346 (4.8)	109 (2.3)	222 (12.0)
Death	307 (4.3)	139 (2.9)	142 (7.7)

Abbreviation: CSI, corticosteroid injection.

^a^ Unless otherwise indicated, data are expressed as No. (%) of patients. Percentages have been rounded and may not total 100.

^b^ Includes African, American Indian or Alaska Native, Chamorro, Guamanian, or Samoan.

^c^ Given in triamcinolone equivalents.

**Table 2.** Location of Corticosteroid Injection^a^

Location	Injection group		
	All (N = 33 864)	Non–high-risk (n = 20 452)	Osteoporosis (n = 10 561)
Joint, No. (%)	17 372 (51.3)	10 417 (50.9)	5295 (50.1)
Large, No.	15 040 (86.6)	9065 (87.0)	4599 (86.8)
Medium, No.	1280 (7.4)	734 (7.1)	395 (7.5)
Small, No.	1052 (6.0)	618 (5.9)	301 (5.7)
Spine region, No. (%)	11 828 (34.9)	7252 (35.5)	3774 (35.7)
Facet, No.	6356 (53.7)	3839 (52.9)	2130 (56.4)
Epidural, No.	4064 (34.4)	2603 (35.9)	1198 (31.7)
Sacroiliac, No.	1408 (11.9)	810 (11.2)	446 (11.8)
Trigger point	1911 (5.6)	1102 (5.4)	654 (6.2)
Nerve block	900 (2.7)	536 (2.6)	304 (2.9)
Tendon or ligament	1398 (4.1)	890 (4.4)	379 (3.6)
Carpal tunnel	366 (1.1)	219 (1.1)	99 (0.9)

^a^ Data are expressed as No. (%) of patients. Percentages have been rounded and may not total 100. Percentages for each subset represent the percentages for their respective location.

Most patients receiving CSIs had a diagnosis of osteoarthritis or bursitis (6361 [88.4%]), and an overlapping portion of these also had at least 1 episode of acute crystalline arthritis during the study (gout or pseudogout [n = 412]). Additionally, 30 patients without osteoarthritis had crystalline arthritis. A further subset of the osteoarthritis group had an episode of acute joint pain (representing conditions such as knee meniscus tear or shoulder adhesive capsulitis [n = 468] and hemarthrosis [n = 28]). In patients without osteoarthritis, 60 had a diagnosis of knee meniscus tear or shoulder adhesive capsulitis, and 2 had a diagnosis of hemarthrosis. A small portion of the overall group (593 [8.2%]) had inflammatory arthritis, mostly rheumatoid arthritis (n = 544) (eTable 4 in Supplement 1).

During the study period, 346 patients (4.8%) had a new fracture, and 149 of these fractures (43.1%) occurred in classic osteoporotic locations (Table 3). The mean time from first CSI to fracture was 329 (range, 2-1422) days (Table 3). In subgroup analysis, there were 4741 patients in the non–high-risk subgroup and 1845 patients in the known osteoporosis subgroup. In the non–high-risk subgroup, only 109 fractures (2.3%) occurred, and 25 of these (22.9%) were in classic osteoporotic fracture locations. In the osteoporosis group, 222 fractures (12.0%) occurred, and 102 of these (45.9%) were in classic osteoporotic fracture locations (Table 3).

**Table 3.** Fracture Characteristics of the Study Cohort

Characteristic	Patient group		
	All (N = 7197)	Non–high-risk (n = 4741)	Osteoporosis (n = 1845)
Fracture, No. (%)	346 (4.8)	109 (2.3)	222 (12.0)
Osteoporotic	149 (43.1)	25 (22.9)	102 (45.9)
Nonosteoporotic	197 (56.9)	84 (77.1)	120 (54.1)
Time from first CSI to fracture, mean No. (range)	329 (2-1422)	376 (2-1318)	313 (4-1443)

Abbreviation: CSI, corticosteroid injection.

The fractures in the whole group were analyzed using cumulative CSI dose quartiles, and no difference was found in the fracture rate between dose quartiles (5.5% [quartile 1] vs 3.6% [quartile 4]; χ^2^ *P* = .87) (Figure). With adjustment for known fracture risk factors, the Cox proportional hazards regression model showed no association of fracture risk across the dose spectrum of cumulative CSIs using 80-mg increments (adjusted HR, 1.04 [95% CI, 0.96-1.11]) (Table 4). There was also no associated risk of fracture in the non–high-risk (adjusted HR, 1.11 [95% CI, 0.98-1.26]) or osteoporosis (adjusted HR, 1.01 [95% CI, 0.90-1.11]) subgroups (Table 4 and eTable 5 in Supplement 1).

**Figure.**
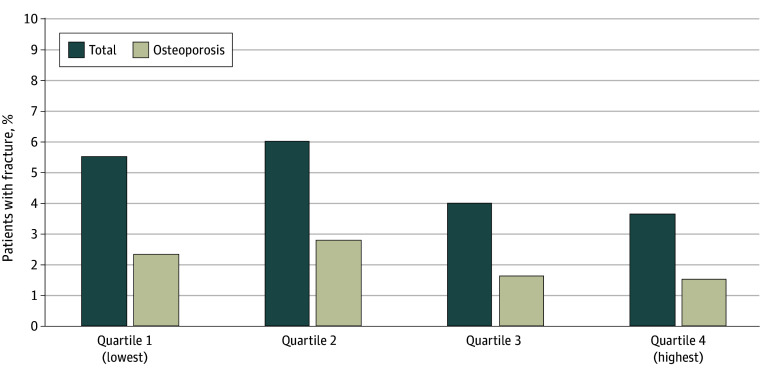
Fracture by Cumulative Dose Quartile Quartile 1 indicates lowest cumulative dose; quartile 4, highest dose. *P* = .87 for all comparisons using χ^2^ test.

**Table 4.** Full Adjusted Cox Proportional Hazards Regression Model With Outcome of Fracture per 80-mg Triamcinolone Equivalent Dose of Corticosteroid Injection

	Patient group, adjusted HR (95% CI)^a^		
	All	Non–high risk	Osteoporosis
Triamcinolone equivalent dose (unit = 80 mg)	1.04 (0.96-1.11)	1.11 (0.98-1.26)	1.01 (0.92-1.11)
Age (unit = 10 y)	1.45 (1.33-1.58)	1.09 (0.94-1.26)	1.33 (1.17-1.52)
Women (compared with men)	1.11 (0.88-1.39)	0.68 (0.46-1.01)	0.77 (0.55 1.08)
Asian, Black, or other (compared with White)	0.59 (0.33-1.05)	0.50 (0.18-1.36)	0.64 (0.30-1.37)
Charlson Comorbidity index (unit = 1.0)	1.07 (1.02 1.12)	1.13 (1.04-1.24)	1.03 (0.97-1.09)
No. of past injections (unit = 1)	0.99 (0.97-1.02)	0.98 (0.92-1.04)	0.98 (0.95-1.01)
Previous fracture (compared with none)	3.26 (2.60-4.09)	3.00 (1.90-4.73)	2.41 (1.84-3.16)

Abbreviation: HR, hazard ratio.

^a^ Adjusted for age, sex, race and ethnicity, Charlson Comorbidity Index score, number of past corticosteroid injections, and previous fracture.

Previous fracture (adjusted HR, 3.26 [95% CI, 2.60-4.09]), age (adjusted HR for unit of 10 years, 1.45 [95% CI, 1.33-1.58]), and Charlson Comorbidity Index (adjusted HR for unit of 1.0, 1.07 [95% CI, 10.2-1.12]) were the only factors that were associated with risk of fracture in the whole group during the study period. In the non–high-risk subgroup, the factors that were associated with higher risk of fracture during the study period were previous fracture (adjusted HR, 3.00 [95% CI, 1.90-4.73]) and Charlson Comorbidity Index (adjusted HR for unit of 1.0, 1.13 [95% CI, 1.04-1.24]). In the osteoporosis subgroup, the factors that were associated with higher risk of fracture during the study period were previous fracture (adjusted HR, 2.41 [95% CI, 1.84-3.16]) and age (adjusted HR for unit of 10 years, 1.33 [95% CI, 1.17-1.52]) (Table 4).

## Discussion

Injected corticosteroids are a common treatment for many painful musculoskeletal conditions, and the possibility of increasing fracture risk with increasing total CSI doses is a concern that could lead clinicians to limit the number of CSIs patients receive. To our knowledge, this study is the first to comprehensively account for all CSI doses a given patient has received in the many clinical settings in which they are performed. The results of our cohort study show that the risk of fracture, either osteoporotic or nonosteoporotic, is not associated with cumulative dose of CSI increases. Furthermore, subgroup analysis showed that there was no association of fracture with higher doses of CSI in patients with osteoporosis, a patient population with a high risk of fracture. Based on these results, treatment of painful conditions with CSIs should not be withheld or delayed owing to concern about fracture risk.

Previous studies^18,19^ have suggested that there is an association between epidural CSIs and decreased bone density, but these studies were small cohort studies with mixed results that did not investigate the fracture risk following epidural corticosteroid injections. A study using Medicare claims data that excluded patients younger than 65 years and patients with previous osteoporotic fracture^20^ showed that large joint CSIs were associated with decreased fractures.

### Strengths and Limitations

Strengths of this study include the large sample size, the specific CSI dose information accounting for the total of CSIs each patient received in all settings, and the clinically relevant outcome of fracture diagnosis. This study also included patients with a preexisting diagnosis of osteoporosis and thus an increased risk of fracture. Furthermore, the long follow-up period of 4 years assessed for delayed CSI outcomes associated with fracture risk. Excluding patients with oral corticosteroid use in amounts that have been correlated with fractures and controlling for other known risks for fracture such as age, previous fracture, and comorbidities also reduced confounding factors in assessing fracture risk from CSIs.

This study also has some limitations. It was a retrospective cohort study in a single community-based, predominantly White population and spanned a relatively small portion of a lifetime. Fracture risk is complex, and it is possible that other confounding variables such as smoking status, alcohol intake, family history of fracture, limited physical activity,^21,22,23^ prolonged lifetime CSI exposure could contribute to overall fracture risk. However, the Charlson Comorbidity Index accounts for connective tissue disease and serves as a surrogate for many other confounding variables. In addition, only clinically apparent fractures were considered, and clinically silent vertebral fractures^24^ would not have been included. This study did not delineate differences in corticosteroid formulation, and there could be differences in systemic outcomes associated with CSIs based on the solubility of the injected corticosteroid. While there was no control group, the wide range of cumulative CSI doses in our cohort (2.7-2140.3 mg of triamcinolone equivalents) accounts for a large spectrum of potential outcomes. The time frame of this study also spans the COVID-19 pandemic, including times when people may have been less likely to seek health care. However, people with fractures of clinical significance would be expected to have had a medical evaluation.

## Conclusions

In this cohort study of cumulative injected corticosteroid dose and risk of subsequent fracture, no association was observed, including in patients with a preexisting diagnosis of osteoporosis. Increasing doses of CSIs were not associated with either osteoporotic or nonosteoporotic fractures. Clinicians should be reassured that frequent CSI is not associated with higher fracture risk and should not withhold these important pain treatments owing to concern for fracture.

## Notes

Supplement 1. **eTable 1.** Corticosteroid Injection *Current Procedural Terminology* Codes **eTable 2.** Fracture Diagnosis Codes **eTable 3.** Lifestyle and Low BMI Distribution per Cumulative Dose Quartile for the Whole Group **eTable 4.** Comorbidities of the Entire Cohort **eTable 5.** Subgroup Analysis of Fracture by Cumulative Dose Quartile

Supplement 2.
Data Sharing Statement
