# Supplementary material for: Corticosteroid Injections and Risk of Fracture
Source: JAMA Netw Open. 2024 May 31;7(5):e2414316. doi: 10.1001/jamanetworkopen.2024.14316 (PMC11143456; doi:10.1001/jamanetworkopen.2024.14316)
Supplement: Supplement 1. — eTable 1. Corticosteroid Injection Current Procedural Terminology Codes eTable 2. Fracture Diagnosis Codes eTable 3. Lifestyle and Low BMI Distribution per Cumulative Dose Quartile for the Whole Group eTable 4. Comorbidities of the Entire Cohort eTable 5. Subgroup Analysis of Fracture by Cumulative Dose Quartile [file jamanetwopen-e2414316-s001.pdf]

Supplementary Online Content

Sytsma TT, Thomas S, Fischer KM, Greenlund LS. Corticosteroid injections and risk of fracture. *JAMA Netw Open*. 2024;7(5):e2414316.
doi:10.1001/jamanetworkopen.2024.14316

eTable 1. Corticosteroid Injection *Current Procedural Terminology* Codes
eTable 2. Fracture Diagnosis Codes
eTable 3. Lifestyle and Low BMI Distribution per Cumulative Dose Quartile for the Whole Group
eTable 4. Comorbidities of the Entire Cohort
eTable 5. Subgroup Analysis of Fracture by Cumulative Dose Quartile

This supplementary material has been provided by the authors to give readers additional information about their work.

© 2024 Sytsma TT et al. *JAMA Network Open*.

eTable 1. Corticosteroid Injection *Current Procedural Terminology* Codes

Joint and bursa injections:

CPT Code	Procedure Description
**20610**	Arthrocentesis, aspiration and/or injection, major joint or bursa (eg, shoulder, hip, knee, subacromial bursa); without ultrasound guidance
**20611**	Arthrocentesis, aspiration and /or injection, major joint or bursa (eg. shoulder, hip, knee, subacromial bursa); with ultrasound guidance
**20605**	Arthrocentesis, aspiration and/or injection, intermediate joint or bursa (eg, temporomandibular, acromioclavicular, wrist, elbow or ankle, olecranon bursa); without ultrasound guidance
**20606**	Arthrocentesis, aspiration and/or injection, intermediate joint or bursa (eg, temporomandibular, acromioclavicular, wrist, elbow or ankle, olecranon bursa); with ultrasound guidance
**20600**	Arthrocentesis, aspiration and/or injection; small joint or bursa (eg, fingers, toes)
**20604**	Arthrocentesis, aspiration and/or injection, small joint or bursa (eg, fingers, toes); with ultrasound guidance
**20550**	Injection of tendon sheath, ligament or muscle
**20551**	Injection of tendon attachment to bone
**20552**	Injection of trigger points 1or 2 muscles
**20553**	Injection of trigger points in 3 or more muscles
**20526**	Injection of carpal tunnel
**64400**	Introduction/Injection of Anesthetic Agent (Nerve Block), Diagnostic or Therapeutic Procedures on the Somatic Nerves
**64405**	Injection, anesthetic agent and/or steroid, greater occipital nerve
**64450**	Injection, anesthetic agent and/or steroid, other peripheral nerve or branch

Epidural steroid Injection for cervical, thoracic and lumbar for both interlaminar and transforaminal:

CPT Code	Procedure Description
**62320**	Injection(s), of diagnostic or therapeutic substance(s) (eg, anesthetic, antispasmodic, opioid, steroid, other solution), not including neurolytic substances, including needle or catheter placement, interlaminar epidural or subarachnoid, **cervical or thoracic**; without imaging guidance
**62321**	**cervical or thoracic** with imaging guidance (ie, fluoroscopy or CT)
**62322**	Injection(s), of diagnostic or therapeutic substance(s) (eg, anesthetic, antispasmodic, opioid, steroid, other solution), not including neurolytic substances, including needle or catheter placement, interlaminar epidural or subarachnoid, **lumbar or sacral (caudal)**; without imaging guidance
**62323**	**lumbar or sacral (caudal)** with imaging guidance (ie, fluoroscopy or CT)

© 2024 Sytsma TT et al. *JAMA Network Open*.

**64479**	Injection(s), anesthetic agent and/or steroid, transforaminal epidural, with imaging guidance (fluoroscopy or CT); **cervical or thoracic**, single level
**64480**	**cervical or thoracic**, each additional level (List separately in addition to code for primary procedure)
**64483**	**lumbar or sacral**, single level
**64484**	**lumbar or sacral**, each additional level (List separately in addition to code for primary procedure)
**62310**	**Cervical or thoracic** epidural injection
**62311**	**Lumbar** epidural injection

Facet injections for cervical, thoracic and lumbar levels

CPT Code	Procedure Description
**64490**	Injection(s), diagnostic or therapeutic agent, paravertebral facet (zygapophyseal) joint (or nerves innervating that joint) with image guidance (fluoroscopy or CT), **cervical or thoracic**; single level
**64491**	**cervical or thoracic** second level (List separately in addition to code for primary procedure)
**64492**	**cervical or thoracic** third and any additional level(s) (List separately in addition to code for primary procedure)
**64493**	Injection(s), diagnostic or therapeutic agent, paravertebral facet (zygapophyseal) joint (or nerves innervating that joint) with image guidance (fluoroscopy or CT), **lumbar or sacral**; single level
**64494**	**lumbar or sacral** second level (List separately in addition to code for primary procedure)
**64495**	**lumbar or sacral** third and any additional level(s) (List separately in addition to code for primary procedure)
**64470**	Injection, anesthetic agent and or steroid, paravertebral facet joint or facet joint nerve; **cervical or thoracic**, single level
**64472**	Injection, anesthetic agent and or steroid, paravertebral facet joint or facet joint nerve; **cervical or thoracic**, each additional level
**64475**	Injection, anesthetic agent and or steroid, paravertebral facet joint or facet joint nerve; **lumbar or sacral**, single level
**64476**	Injection, anesthetic agent and or steroid, paravertebral facet joint or facet joint nerve; **lumbar or sacral**, each additional level

Sacroiliac injections

CPT Code	Procedure Description
**27096**	Injection procedure for **sacroiliac joint**, anesthetic/steroid, with image guidance (fluoroscopy or CT) including arthrography when performed

© 2024 Sytsma TT et al. *JAMA Network Open*.

Paravertebral blocks

CPT Code	Procedure Description
**64461**<br>**64462**	Introduction/Injection of Anesthetic Agent (Nerve Block), Diagnostic or Therapeutic Procedures on the Extracranial Nerves, Peripheral.

© 2024 Sytsma TT et al. *JAMA Network Open*.

eTable 2. Fracture Diagnosis Codes

Fracture ICD 10 and ICD 9 codes:

ICD 9 Code	Diagnosis
**733.13**	Vertebral fracture
**805.x**	Fracture of vertebral column
**806.4**	Lumbar spine fracture
**807.x**	Rib fracture
**808.x**	Pelvic Fracture
**810.x**	Clavicle fracture
**811.x**	Scapula Fracture
**812.x**	Humerus fracture
**813.x**	Radius Fracture
**814.x**	Wrist Fracture
**815.x**	Metacarpal Fracture
**816.x**	Finger fracture
**817.x**	Hand fractures
**820.x**	Femur fracture
**821.x**	Femur fracture
**822.x**	Patellar Fracture
**823.x**	Fibular/Tibia fracture
**824.x**	Ankle fracture
**825.x**	Metatarsal fracture
**826.x**	Foot fracture
**829.x**	Unspecified closed fracture
**ICD 10 Code**	**Diagnosis**
**M80**	Unspecified osteoporosis fracture
**S12.x**	Cervical vertebral fracture
**S22.0**	Thoracic vertebral fracture
**S22.3**	Rib fracture
**S32.0**	Lumbar vertebral fracture
**S32.5**	Pubic fracture
**S32.8**	Pelvic fracture
**S42.x**	Shoulder or arm fracture
**S62.x**	Hand or wrist fracture
**S72.x**	Femur fracture

© 2024 Sytsma TT et al. *JAMA Network Open*.

**S82.x**	Lower leg fracture
**M80.00**	Unspecified osteoporosis fracture
**M80.05**	Hip/femur fracture
**M80.80**	Unspecified osteoporosis fracture
**M80.83**	Forearm fracture
**M80.88**	Vertebral fracture
**M97.01**	Periprosthetic hip fracture
**M97.31**	Periprosthetic arm fracture
**M97.42**	Periprosthetic arm fracture

Osteoporotic Fracture ICD 10 and ICD 9 codes:

ICD 9 Code	Diagnosis
**733.13**	Vertebral fracture
**805.x**	Fracture of vertebral column
**806.4**	Lumbar spine fracture
**813.x**	Radius Fracture
**814.x**	Wrist Fracture
**ICD 10 Code**	**Diagnosis**
**M80**	Unspecified osteoporosis fracture
**S12.x**	Cervical vertebral fracture
**S22.0**	Thoracic vertebral fracture
**S32.0**	Lumbar vertebral fracture
**M80.00**	Unspecified osteoporosis fracture
**M80.05**	Hip/femur fracture
**M80.80**	Unspecified osteoporosis fracture
**M80.83**	Forearm fracture
**M80.88**	Vertebral fracture
**M97.01**	Periprosthetic hip fracture
**M97.31**	Periprosthetic arm fracture
**M97.42**	Periprosthetic arm fracture

© 2024 Sytsma TT et al. *JAMA Network Open*.

eTable 3. Lifestyle and Low BMI Distribution per Cumulative Dose Quartile for the Whole Group

	% LOW BMI (<20 KG/M^2)	% CURRENT SMOKER	% ALCOHOL MULTIPLE TIMES A WEEK
**HIGHEST CSI QUARTILE (N=1839)**	6.8	5.4	17.5
**(N=1645)**	7.1	5.9	16.7
**(N=1528)**	7.1	5.6	19.9
**LOWEST CSI QUARTILE (N=2185)**	7.7	5.3	18.6
**CHI-SQUARE P-VALUE**	p>0.79	p>0.86	p>0.12

eTable 4. Comorbidities of the Entire Cohort

COMORBIDITY	% WHOLE GROUP
**OSTEOPOROSIS**	25.6
**RHEUMATOID ARTHRITIS**	7.6
**HEART FAILURE**	9.9
**OTHER AUTOIMMUNE**	6.9
**INFLAMMATORY BOWEL DISEASE**	2.4
**HYPERTENSION**	22.3
**RENAL DISEASE**	34.3
**DIABETES**	27.6
**CORONARY ARTERY DISEASE**	68.2
**CHRONIC OBSTRUCTIVE PULMONARY DISEASE**	11.0
**ME/CFS**	2.2

ME/CFS=myalgic encelphalomyelitis/chronic fatigue syndrome

© 2024 Sytsma TT et al. *JAMA Network Open*.

eTable 5. Subgroup Analysis of Fracture by Cumulative Dose Quartile

	Triam Q1 (N=1506)	Triam Q2 (N=1042)	Triam Q3 (N=1106)	Triam Q4 (N=1087)	Total (N=4741)	P-value
**Non-High Risk group Fracture, n (%)**	39 (2.6)	34 (3.2)	20 (1.8)	16 (1.5)	109 (2.3)	0.46[1]

	Triam Q1 (N=515)	Triam Q2 (N=356)	Triam Q3 (N=395)	Triam Q4 (N=579)	Total (N=1845)	P-value
**Osteoporosis group Fracture, n (%)**	80 (15.5)	54 (15.1)	41 (10.4)	47 (8.1)	222 (12.0)	0.49[1]

**Whole group Fracture, n (%)**	Triam Q1 (N=2185)	Triam Q2 (N=1528)	Triam Q3 (N=1645)	Triam Q4 (N=1839)	Total (N=7197)	P-value
	121 (5.5)	92 (6.0)	66 (4.0)	67 (3.6)	346 (4.8)	0.87[1]

[1]Chi-Square p-value

© 2024 Sytsma TT et al. *JAMA Network Open*.
