# Supplementary material for: Corticosteroid Injections and Risk of Fracture
Source: JAMA Netw Open. 2024 May 31;7(5):e2414316. doi: 10.1001/jamanetworkopen.2024.14316 (PMC11143456; doi:10.1001/jamanetworkopen.2024.14316)
Supplement: Supplement 2. — Data Sharing Statement [file jamanetwopen-e2414316-s002.pdf]

Data Sharing Statement

Sytsma. Corticosteroid Injections and Risk of Fracture. *JAMA Netw Open*. Published May 31, 2024. doi:10.1001/jamanetworkopen.2024.14316

Data
Data available: No

Additional Information
Explanation for why data not available: Data is available upon reasonable request
